# Water filtration by endobenthic sandprawns enhances resilience against eutrophication under experimental global change conditions

**DOI:** 10.1038/s41598-023-46168-y

**Published:** 2023-11-04

**Authors:** C. M. Thomas, C. de Cerff, G. A. V. Maniel, A. E. Oyatoye, E. Rocke, H. G. Marco, D. Pillay

**Affiliations:** 1 Department of Biological Sciences, Marine and Antarctic Research Centre for Innovation and Sustainability, University of Cape Town, Cape Town, 7701 South Africa; 2 Marine and Freshwater Research Centre, Atlantic Technological University, Galway, Ireland; 3 Laboratoire de Biologie des Organismes et Ecosystèmes Aquatiques (UMR 8067 BOREA), Muséum national d’Histoire naturelle, 61 rue Buffon, 75005 Paris, France

**Keywords:** Ecosystem services, Urban ecology, Wetlands ecology

## Abstract

Identifying processes that confer resilience against global change is a scientific challenge but is central to managing ecosystem functionality in future. Detecting resilience-enhancing mechanisms is especially relevant in coastal ecosystems, where multi-stressor interactions can drive degradation over time. Here, we quantify the resilience-conferring potential of endobenthic sandprawns against eutrophication, including under high temperatures. We show using a global change mesocosm experiment that sandprawn presence was associated with declines in phytoplankton biomass, particularly under eutrophic conditions, where sandprawns reduced phytoplankton biomass by approximately 74% and prevented a shift to extreme eutrophy. Eutrophic waters were nanophytoplankton-dominated, but sandprawn presence countered this, resulting in even contributions of pico- and nanophytoplankton. Our findings highlight the potential for sandprawns to increase resilience against eutrophication by limiting phytoplankton blooms, preventing extreme eutrophy and counteracting nanophytoplankton dominance. Incorporating endobenthic crustaceans into resilience-based management practices can assist in arresting future water quality declines in coastal ecosystems.

## Introduction

Rapid urbanisation, economic development and habitat degradation are features of coastal ecosystems in the Anthropocene^1–3^. High human densities, increasing population growth and rising demand for habitation, industry, and recreation, have intensified anthropogenic pressures on coastal ecosystems, with climate change further heightening ecological stress^3–5^. Of concern is the potential for interactions between multiple stressors and intrinsic ecological processes to erode ecosystem resilience and drive shifts to undesirable states^6^. Given the economic costs involved in environmental remediation from degraded states, preventing undesirable state shifts is an important objective of ecosystem management^7–9^. Additionally, effecting ecosystem recovery may require stressors to be reduced to levels that predated degradation, but achieving this is difficult^10^. Preventing ecosystem shifts to undesirable states is additionally hampered by (1) knowledge limitations concerning processes that oppose ecosystem shifts to undesirable states^6^ and (2) uncertainty regarding how climate change and anthropogenic stressors interact to impact ecosystems^4^, including resilience-conferring processes.

Eutrophication is a pervasive and severe stressor in coastal zones globally, given its ability to induce large-scale ecosystem shifts to degraded states^11–13^. Specifically, excessive nutrient inputs into water bodies trigger proliferation of opportunistic algae and phytoplankton blooms, which in turn can initiate toxic algal growth, hypoxia/anoxia, aquatic macrophyte losses, food-web alterations, and consumer mass-mortality^11–13^. Local-scale management of biological processes that can assist in resisting and/or reversing switches to algal-dominated states has been identified as a counter-measure to coastal eutrophication^13,14^. Restoration of filter-feeder and vegetated biotopes for example, have been proposed to limit the development of eutrophication symptoms through phytoplankton consumption and nutrient uptake^13,14^. However, such management strategies are complicated by the fact that eutrophication, like other anthropogenic stressors, does not operate independently of global change processes^3^. The implication, therefore, is that local-scale management of eutrophication through interventions involving biological processes requires knowledge of their resilience to future global change scenarios.

Endobenthic organisms have rarely featured in eutrophication mitigation strategies for coastal ecosystems. In contrast, vegetated ecosystems and filter-feeder assemblages have been more prominent as natural counter measures for eutrophication^13,14^. Recent research, however, has shown that some endobenthic deposit-feeding crustaceans (sandprawns), despite their functional designation, are capable of exerting top-down control on phytoplankton^15^. Specifically, sandprawn presence was shown to reduce phytoplankton biomass by almost 50% in experimental mesocosms, with similar effects recorded in situ. Bi-directional water pumping during ventilation/irrigation was hypothesized to increase phytoplankton entrapment onto burrow walls, thereby reducing phytoplankton biomass in the water column^15^. This led to the suggestion that below-ground burrow superstructures of sandprawns, and similar endobenthic organisms, may function as biological filtration systems that can increase resilience against coastal eutrophication^15^. However, the robustness of sandprawn water filtration to future global change conditions is unknown, even though addressing this issue is of both scientific and management relevance.

Extremes of coastal eutrophication and warming are two global change scenarios that have the potential to weaken the water filtration function provided by sandprawns and similar endobenthic organisms, based on research conducted on other consumers. For ectothermic consumers, increasing water temperature up to thermal optima can increase metabolic demand, resource consumption and top-down impact on trophic resources^16–18^. Beyond thermal optima though, rising temperature may impair consumer physiology, increase mortality and alter distribution^19,20^. Similarly, at high levels of eutrophication, oxygen depletion, pathogenic microbes, harmful algae and released toxins may increase the incidence of disease or mortality in consumers, negating their contribution to ecosystem resilience^11,12,20,21^. Thus, impaired physiology and health, in addition to greater mortality at high temperature and eutrophication levels, may have the effect of weakening total resource consumption and hence top-down impact. Shifts to phytoplankton taxa that are less edible in response to nutrient enrichment and high temperature can also potentially weaken top-down impacts^21,22^. To complicate matters, coastal warming and eutrophication can directly impact phytoplankton through metabolic changes^16,23,24^. Based on metabolic theory, increasing temperature is expected to reduce phytoplankton biomass due to rising temperature increasing demand for rate-limiting resources. However, if resources do not increase in quantity, they become insufficient to support phytoplankton metabolic requirements^24^. However, if concentrations of limiting nutrients were to increase in parallel (as with eutrophication), then warming may elicit increases in phytoplankton biomass, due to metabolic demands of phytoplankton being met, though this may result in phytoplankton shifts to taxa that are less palatable^23^. In this scenario, phytoplankton biomass may rise to levels that cannot be controlled by sandprawns, given that they are not specialist filter-feeders^25^, which are likely to be more efficient and selective in phytoplankton removal.

The effects of coastal warming, eutrophication and top-down processes on phytoplankton can thus be complex, necessitating a thorough understanding of these effects to predict and manage future changes in coastal ecosystems. We therefore addressed this issue in the context of South African temporarily closed estuaries, which account for 71% of estuaries in the country^26,27^. Likely due to the semi-arid climate, these systems periodically close-off from oceans^26,27^, although this may be exacerbated by drought and increasing water abstraction for human needs. Temporarily closed estuaries also occur in Australia, south-eastern Brazil and Uruguay, south-western India, Sri Lanka^27^, Portugal, as well as along the coastlines of California and Texas in the United States of America^21^. Consistent with global trends^11,12^, eutrophication is a key process impacting South African estuaries, but the consequences of inorganic nutrient inputs (such as from fertilisers) are compounded by organic matter inputs from wastewater treatment works that lack operational skills and resources for maintenance and upgrades^13^. Wastewater treatment facilities are typically overloaded and malfunctioning, resulting in spillage of poorly treated wastewater into estuaries^13^. Additionally, the shallow depths (< 1.5 m) of intermittently closed estuaries^28^ may increase the risk to rising air temperatures relative to deeper permanently open systems, as has been postulated for shallow lakes^29^ ^and refs therein^. For South Africa, air temperature rose by 0.03° C/year over the period 1980–2014^30^, and global temperature is predicted to rise by 4 °C on average by 2100^31^.

Sandprawns (*Kraussillichirus kraussi*; Crustacea: Axiidea) are abundant in benthic ecosystems in estuaries locally, including those that have been closed to the ocean for several years^32^. Sandprawns have a wide distribution across the Southern African coastline from Namibia (cool-temperate west coast) to Mozambique (sub-tropical east coast^32^) and belong to a globally distributed group of crustaceans (formerly Thalassinidea) that are renowned as highly proficient endobenthic ecosystem engineers^25,33^. These organisms occur in dense aggregations that cover large areas (several km), burrow to depths greater than 1 m and manipulate sediment at high rates^25,33^ (12.14 kg/m^2^/day or 4.4 ton/m^2^/year for *K. kraussi*^34^). While much is known about endobenthic crustaceans as sedimentary ecosystem engineers, little is understood about their role as top-down regulators of phytoplankton assemblages or their potential to increase resilience against eutrophication. In the current study, we used a factorial mesocosm experiment to test the effects of sandprawn density, eutrophication and warming on phytoplankton assemblages. We firstly tested whether sandprawn water-filtration was impaired under eutrophic and warming conditions to assess filtration robustness to expected nutrient loading and thermal changes in future. We were especially interested in determining whether sandprawn filtration could counter phytoplankton blooms associated with high levels of eutrophication to understand the resilience conferring potential of sandprawns to upper limits of this stressor. We also assessed whether eutrophication and warming acted antagonistically, additively, or synergistically to impact phytoplankton. Lastly, we measured phytoplankton responses in terms of biomass (or abundance) and size, to understand how top-down sandprawn effects, eutrophication and warming interacted to alter both phytoplankton quantity and traits.

## Materials and methods

### Study site

**Figure 1:**
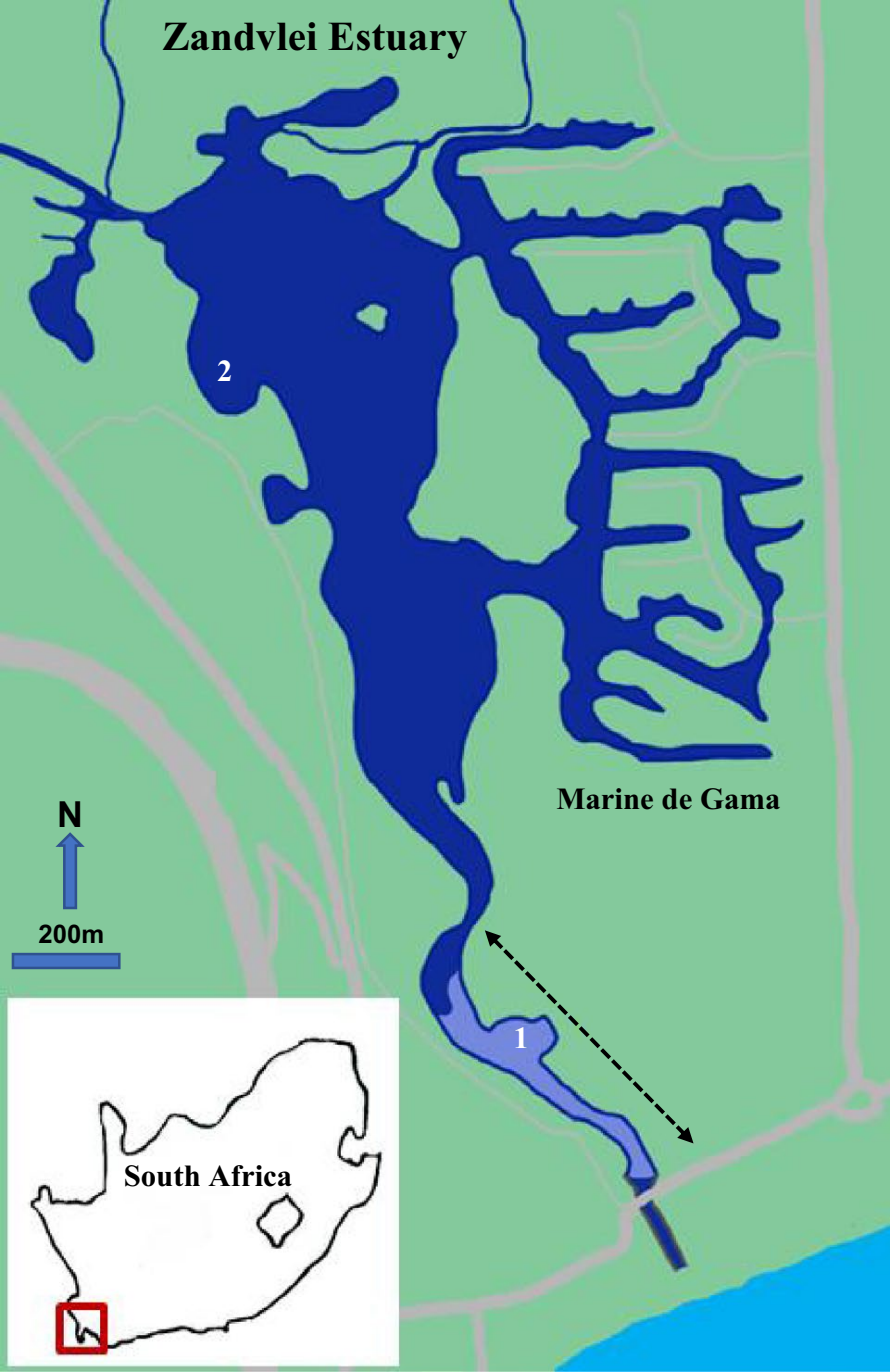
Map of the Zandvlei Estuary showing its location within South Africa (inset). The dashed arrow indicates the extent of the sandprawn habitat in the lower reaches. Sandprawns, sediment and mesotrophic water were collected from Site 1 and eutrophic water was collected from Site 2. Modified from Venter et al. (2020)^15^.

The large temporarily closed Zandvlei Estuary (34°05 'S; 18°28 'E) was the focal system of study, from which materials for the experiment were collected. The system is also an ideal model coastal ecosystem to understand multiple stressor effects on ecological processes, given that it is highly modified anthropogenically, yet remains a functionally important urban estuary. The system is located in False Bay, in the City of Cape Town, South Africa (Fig. 1), is roughly 2.5 km in length and has a mean depth of 1.4 m^35,36^. The system has been canalized in the lower reaches and a weir constructed for mouth management. A marina has been constructed on its eastern shores and periodic dredging of the main channel occurs in the lower reaches^36^. The water level in the estuary is mechanically regulated^35^ and the mouth is opened once per month (spring high tide) during summer (low rainfall) but is kept open during winter (high rainfall)^15,36^. Riverine inputs that include discharge from urbanized and industrialized areas have resulted in the system being classified as eutrophic^35^, but this is likely exacerbated by sewage spills associated with ailing infrastructure. Harmful algal blooms have been recorded, including that of toxic golden algae *Prymnesium parvum*, which has been linked to fish mortality in the system^36^. The lower reaches of the estuary are occupied by dense populations (114/m^2^–240/m^2^) of the sandprawn *Kraussillichirus kraussi*^15^ (Supplementary Fig. S1).

### Experimental design

A 16-day (14–29 November 2020) indoor mesocosm experiment was conducted at the aquarium facilities at the University of Cape Town (UCT), Department of Biological Sciences, to quantify the effects of eutrophication, warming and sandprawn densities on phytoplankton biomass and traits. Air temperature was set to 15 °C and lighting on a 14-h day/10-h night cycle. Glass (8 mm thick) mesocosms (height: 600 mm, length: 300 mm, width: 300 mm) were used to create 36 independent experimental units for the study. The experiment involved manipulating (1) sandprawn density (three levels, 0% natural density = no sandprawns; 50% natural density = 9 sandprawns per mesocosm; 100% natural density = 18 sandprawns per mesocosm), (2) eutrophication (two levels, mesotrophic (chl-*a* concentration ~ 10 µg/L^37,38^) versus eutrophic (chl-*a* concentration ~ 20 µg/L^37,38^) waters) and (3) temperature (two levels, low ~ 14 °C and high ~ 29 °C), with n = 3. Sandprawn numbers used per mesocosm was determined by scaling down maximum sandprawn numbers reported for South African estuaries and lagoons (~ 200 individuals/m^2^)^34^ to the area of each mesocosm.

Naturally occurring waters were used in the experiment to test ecological responses to mesotrophic and eutrophic conditions. The advantage of this approach is that it allowed us to include potential effects of environmental pathogens/toxins in assessing the robustness of sandprawn filtration effects, particularly under eutrophic conditions. Eutrophic water (chl-*a* > 15 µg/L; based on classification for semi-arid climates^37,38^), salinity = 10, temperature = 19 °C) was collected from the upper reaches of the estuary (Site 2, Fig. 1). Mesotrophic water (chl-*a* < 15 µg/L, salinity = 29, temperature = 18 °C) was collected from the lower reaches of the system (Site 1, Fig. 1), along with sediment and sandprawns. Eutrophic and mesotrophic waters were filtered separately (200 µm mesh) into 100 L vats and homogenized. Salinity of the water for each trophic treatment was standardized to 29 using marine salt (Aquamedic). Estuarine sediment was sieved (2 mm mesh) before being added to each mesocosm (depth = 25 cm). This was followed by the addition of either eutrophic or mesotrophic water (depth = 25 cm) to each mesocosm. Water within each mesocosms was aerated and allowed to settle for 24 h before recording initial (Day 0) water quality data. Sandprawns were thereafter introduced according to density designations. Non-gravid sandprawns (> 40 mm; rostrum to telson) were collected using stainless steel prawn pumps (length = 900 mm, diameter = 50 mm), placed in loose, moistened layers of newspaper, transported to the aquarium facility and left to acclimatise for 2–3 h prior to being added to mesocosms.

High water temperature treatments were established using aquarium heaters (set at 30 °C). This temperature was based on maximum temperatures recorded in the Zandvlei Estuary at sites close to the sandprawn biotope (25.5–26 °C)^35^ plus a predicted 4 °C rise in global temperature by the year 2100^31^. This temperature level allowed us to assess the robustness of sandprawn water-filtration to predicted high temperatures at the end of the century, as well as its potential interaction with eutrophication. Sandprawns allocated to high-temperature treatments were first placed in water-filled bowls in mesocosm water to allow gradual acclimatization to mesocosm temperature. For the duration of the experiment, the number of burrow openings in each mesocosm was monitored. Where number of burrow openings decreased, sandprawns were added to maintain density designations^15^.

### Data collection

Water column temperature, salinity, pH, turbidity and dissolved oxygen were measured per mesocosm with a multiprobe (YSI 650 MPI) on Day 0 and every three days thereafter until the experiment terminated. Nutrient concentrations (ammonium (NH_4_^+^), nitrite (NO_2_^-^), nitrate (NO_3_^-^), phosphate (PO_4_^3-^)) were measured per mesocosm on Day 0 and every week thereafter from 40 mL surface water that was collected using a syringe fitted with a flexible tube (diameter = 5 mm). Water samples were stored at -20 °C until analysis using a multiparameter photometer (Hanna Instruments HI 83,203).

Phytoplankton biomass (as chl-*a* concentration) was measured on Day 0 and every three days thereafter until the experiment terminated. Two 2 mL water samples were collected from each mesocosm (depth = 5 cm) and pooled, from which chl-*a* concentration was determined fluorometrically (Turner Designs Trilogy). Relative phytoplankton cell sizes and absolute abundance thereof were determined using flow cytometry (Faculty of Health Sciences, University of Cape Town). The BD LSR II flow cytometer used in this study was fitted with an air-cooled argon-ion laser (488 mm, 20 mW). Forward-scatter (FSC) was detected by a photodiode detector with a 488/10 bandpass filter and provided information on relative cell size. Side scatter (SSC) was detected by a photomultiplier tube (PMT) with a 488/10 bandpass filter and provided data on cell granularity. Fluorescent beads (AccuCount Fluorescent Particles, Spherotech, Lake Forest, IL, USA) with a standard concentration were run to determine absolute cell counts by comparing cellular events to bead events^39,40^. Additionally, 0.88 μm sized fluorescent beads were used as a standard reference to determine the relative cell sizes present in the sample. Phytoplankton is naturally auto-fluorescent due to photosynthetic pigments and can be identified by its unique fluorescence emission spectra^40^. Picoplankton and nanoplankton were measured by their emission signals on the orange phycoerythrin (PE): 585/42 bandpass vs. red (PC: 661/16 band pass) fluorescence signals.

At the start of the experiment (Day 0) and every three days thereafter, three water samples (depth = 5 cm) were randomly collected from each mesocosm using a syringe and pooled (to account for within-mesocosm variability) into 2 mL cryovials for flow cytometry analysis. Samples were stored at 4 °C in darkness and analysed on the day of collection. Aliquots of 1 mL were drawn from the pooled sample, vortexed and analysed at a low flow rate with a threshold of ~ 100 000 events or ~ 10 min per sample using the BD LSRII. Flow cytometry data were analysed by gating populations using FlowJo Software^41,42^ (Version 10.7.2). Population counts of nanophytoplankton and picophytoplankton were converted into concentrations (cells/mL) on Microsoft Excel V16.5 using a standard formula incorporating flow rate and run time of each sample. The proportion of nano- and picophytoplankton was calculated for Day 0 and compared with a mean of values from Day 6 until the experiment terminated on Day 15. At the end of the experiment, sandprawns were carefully extracted from the mesocosms and returned to the Zandvlei Estuary.

### Data analysis

All data analyses were performed using the data analysis platform R (v4.1.2, 2021^43^). Linear Mixed-Effects Models (LMEMs) were fitted by restricted maximum likelihood (REML) estimation using the *‘lme4’* package^44^ to determine the effects of sandprawn density, eutrophication, and temperature on biotic (chl-*a,* picoplankton and nanoplankton concentrations) and abiotic response variables (physico-chemical and inorganic nutrient data). For all LMEMs, time and mesocosm ID were included as random factors since samples were not temporally independent^15^. Model fits were graphically evaluated using histograms, quantile–quantile (Q–Q) plots and plots of residuals against predicted values to check for normality and homogeneity of variance^45^. Where model assumptions were violated, models were re-fitted using transformed data. The ‘Anova’ function in the *‘car´* package was applied to models to determine the significance of main and interactive effects of predictors, given that significance levels were not provided in model outputs for fixed effects^46^.

### Ethical approval

Experiments were conducted in accordance with guidelines of the University of Cape Town. Approval of the research was granted by the University of Cape Town, Science Faculty Animal Ethics Committee (approval number 2018/v10/DP).

## Results

Variation in pelagic abiotic conditions among sandprawn densities was negligible; where statistically significant effects were detected, variance was minimal (Table 1; Supplementary Table S1). Variability in salinity was explained by all main predictors and the sandprawn × eutrophication interaction. Salinity levels were reduced marginally in the presence of sandprawn in mesotrophic treatments, particularly towards the end of the experiment (Table 1). At most though, for any given day of sampling, salinity varied between 36.6 ± 1.1 and 33.6 ± 1.6 SE across increasing sandprawn densities (Supplementary Table S1). Variance in pH was explained by temperature, with values increasing by 0.3 to 0.4 units with rising temperature (Table 1).

**Table 1:** Results of type II Wald Chi-Square analyses testing the main and interactive effects of predictor variables (sandprawn density, eutrophication, temperature) on the abiotic environmental response variables.

Predictor	Abiotic variable	χ^2^	df	*p* value
SP	Dissolved oxygen	2.11	2	0.35
	pH	0.78	2	0.68
	Salinity	**8.95**	**2**	**0.01**
	Temperature	0.593	2	0.743
	Turbidity	1.16	2	0.56
E	Dissolved oxygen	0.10	1	0.75
	pH	0.01	1	0.92
	Salinity	**11.43**	**1**	***p *< 0.001**
	Temperature	1.38	1	0.24
	Turbidity	1.98	1	0.16
T	Dissolved oxygen	**145.60**	**1**	***p *< 0.0001**
	pH	**85.26**	**1**	***p *< 0.0001**
	Salinity	**145.85**	**1**	***p *< 0.0001**
	Temperature	**933.32**	**1**	***p *< 0.0001**
	Turbidity	**5.25**	**1**	**0.02**
SP × E	Dissolved oxygen	0.44	2	0.80
	pH	1.42	2	0.49
	Salinity	**6.05**	**2**	**0.049**
	Temperature	0.52	2	0.77
	Turbidity	0.01	2	0.99
SP × T	Dissolved oxygen	0.85	2	0.65
	pH	5.82	2	0.05
	Salinity	3.16	2	0.20
	Temperature	1.22	2	0.54
	Turbidity	2.93	2	0.231
SP × E × T	Dissolved oxygen	4.88	2	0.09
	pH	0.52	2	0.77
	Salinity	1.94	2	0.38
	Temperature	1.00	2	0.60
	Turbidity	1.57	2	0.46

Statistically significant outcomes are displayed in bold.

*SP* Sandprawn density, *E* Eutrophication, *T* Temperature. *χ*^*2*^ Test statistic, *df* Degrees of freedom, *p value* Significance level.

Water temperature ranged between 14 ± 0.1 SE and 16.5 °C ± 1.2 SE and 26.8 ± 0.4 SE and 29.7 °C ± 0.5 SE in low and high temperature treatments, respectively and corresponded with a priori designations of levels in the temperature treatment. Salinity in high temperature mesocosm increased over the course of the experiment by 5 to 6, but this trend was not evident at low temperatures (Supplementary Table S1). A similar trend was recorded for dissolved oxygen in high temperature treatments, with percentage saturation increasing to around 100% from starting conditions of approximately 95%, whereas oxygen levels in low temperature treatments seldom varied by more than 2% (Supplementary Table S1).

Pelagic inorganic nutrient levels were unaffected by sandprawn density treatments and eutrophication, but variance in NH_4_^+^, NO_2_^-^ and PO_4_^3-^ levels was explained by temperature (Table 2). Generally, NH_4_^+^ and NO_2_^-^ levels were greater in the high temperature treatments, especially towards the end of the experiment (Supplementary Table S2). Lower PO_4_^3-^ concentrations were recorded under high temperature, particularly in eutrophic conditions (Supplementary Table S2).

**Table 2:** Results of type II Wald Chi-Square analyses testing the main and interactive effects of predictor variables (sandprawn density, eutrophication, temperature) on inorganic nutrient concentrations.

Predictor	Inorganic nutrient	χ^2^	df	*p* value
SP	Phosphate (PO_4_^3-^)	0.10	2	0.95
	Ammonium (NH_4_^+^)	0.92	2	0.63
	Nitrate (NO_3_^-^)	4.11	2	0.13
	Nitrite (NO_2_^-^)	3.24	2	0.20
E	Phosphate (PO_4_^3-^)	0.022	1	0.88
	Ammonium (NH_4_^+^)	0.04	1	0.84
	Nitrate (NO_3_^-^)	0.23	1	0.63
	Nitrite (NO_2_^-^)	0.85	1	0.36
T	**Phosphate (PO**_**4**_^**3-**^**)**	**9.57**	1	**0.002**
	**Ammonium (NH**_**4**_^**+**^**)**	**8.96**	**1**	**0.003**
	Nitrate (NO_3_^-^)	0.33	1	0.57
	**Nitrite (NO**_**2**_^**-**^**)**	**61.9**	**1**	** < 0.0001**
SP × E	Phosphate (PO_4_^3-^)	0.81	2	0.67
	Ammonium (NH_4_^+^)	0.44	2	0.80
	Nitrate (NO_3_^-^)	0.25	2	0.88
	Nitrite (NO_2_^-^)	2.00	2	0.37
SP × T	Phosphate (PO_4_^3-^)	0.70	2	0.68
	Ammonium (NH_4_^+^)	0.66	2	0.72
	Nitrate (NO_3_^-^)	3.07	2	0.22
	Nitrite (NO_2_^-^)	6.32	2	0.04
SP × E × T	Phosphate (PO_4_^3-^)	0.85	2	0.65
	Ammonium (NH_4_^+^)	1.07	2	0.59
	Nitrate (NO_3_^-^)	0.05	2	0.97
	Nitrite (NO_2_^-^)	2.30	2	0.32

Statistically significant outcomes are displayed in bold.

*SP* Sandprawn density, *E* Eutrophication, *T* Temperature. *χ*^*2*^ Test statistic, *df* Degrees of freedom, *p value* Significance level.

**Figure 2:**
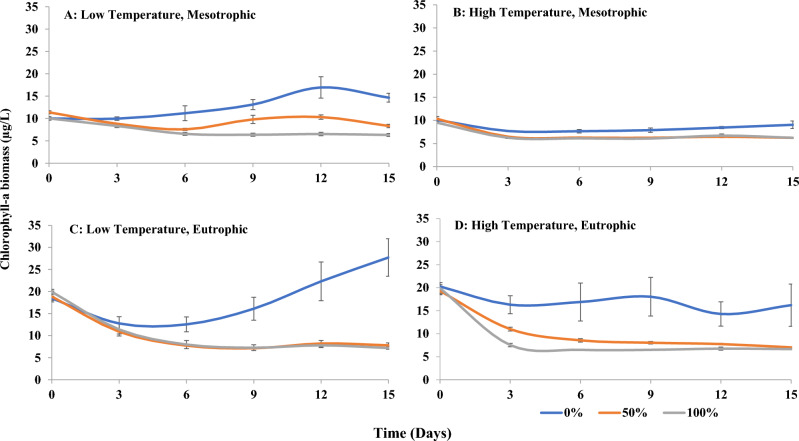
Spatio-temporal variability in chl-*a* biomass (mean ± SE) in (**A**) (low temperature, mesotrophic), (**B**) (high temperature, mesotrophic), (**C**) (low temperature, eutrophic) and (**D**) (high temperature, eutrophic) mesocosms at varying sandprawn densities (0%, 50% and 100%).

Phytoplankton biomass was influenced by all main predictor variables, and except for the sandprawn density × temperature interaction, by all combinations of predictor interactions (Table 3). Phytoplankton biomass was consistently reduced in the presence of sandprawns from Day 3 until the termination of the experiment, irrespective of eutrophication or temperature treatment, relative to controls (Fig. 2). By the end of the experiment, phytoplankton biomass was similar in the presence of sandprawns across all thermal and trophic state treatments (Fig. 2). The greatest decline in phytoplankton biomass in mesocosms with sandprawns relative to controls was recorded in under low temperature, eutrophic conditions (74% by termination; Fig. 2C). Smaller phytoplankton biomass declines in mesocosms containing sandprawns was recorded under low temperature, mesotrophic (43%; Fig. 2A) and high temperature, eutrophic conditions (59%; Fig. 2D) by the end of the experiment, relative to controls. The smallest phytoplankton biomass declines in mesocosms containing sandprawns was recorded under high temperature, mesotrophic conditions (32%; Fig. 2B) relative to controls at the termination of the experiment. By the end of the experiment, phytoplankton biomass in control mesocosms at low temperatures increased beyond starting levels on Day 0, increasing by approximately 32% in mesotrophic (Fig. 2A) and eutrophic (Fig. 2C) treatments. In contrast, at high temperatures, phytoplankton biomass in controls were similar to start levels, with no evidence of an increase over time (Fig. 2B,D).

**Table 3:** Results of type II Wald Chi-Square analyses testing the effects of the predictor variables (sandprawn density, eutrophication, temperature) on phytoplankton response variables.

Plankton variable	χ^2^	df	*p* value
SP			
** Chl-*a***	**197.18**	**2**	** < 0.0001**
** Nanoplankton**	**55.97**	**2**	** < 0.0001**
Picoplankton	5.82	2	0.05
E			
** Chl-*a***	**74.52**	**1**	** < 0.0001**
** Nanoplankton**	**81.40**	**1**	** < 0.0001**
Picoplankton	3.18	1	0.07
T			
** Chl-*a***	**31.32**	**1**	** < 0.0001**
Nanoplankton	2.33	1	0.13
Picoplankton	1.16	1	0.28
SP × E			
** Chl-*a***	**8.06**	**2**	**0.02**
** Nanoplankton**	**9.12**	**2**	**0.01**
Picoplankton	2.44	2	0.29
SP × T			
Chl-*a*	0.54	2	0.76
** Nanoplankton**	**7.85**	**2**	**0.02**
Picoplankton	3.31	2	0.19
SP × E × T			
** Chl-*a***	**12.41**	**2**	**0.002**
Nanoplankton	0.62	2	0.73
Picoplankton	1.66	2	0.44

Statistically significant outcomes are displayed in bold.

*SP* Sandprawn density, *E* Eutrophication, *T* Temperature. *χ*^*2*^ Test statistic, *df* Degrees of freedom, *p value* Significance level.

**Figure 3:**
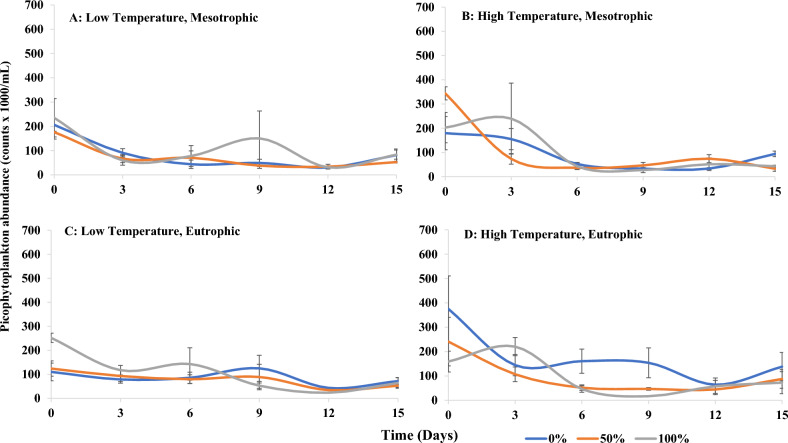
Spatio-temporal variability in picophytoplankton abundance (mean ± SE) in (**A**) (low temperature, mesotrophic), (**B**) (high temperature, mesotrophic), (**C**) (low temperature, eutrophic) and (**D**) (high temperature, eutrophic) mesocosms at varying sandprawn densities (0%, 50% and 100%).

**Figure 4:**
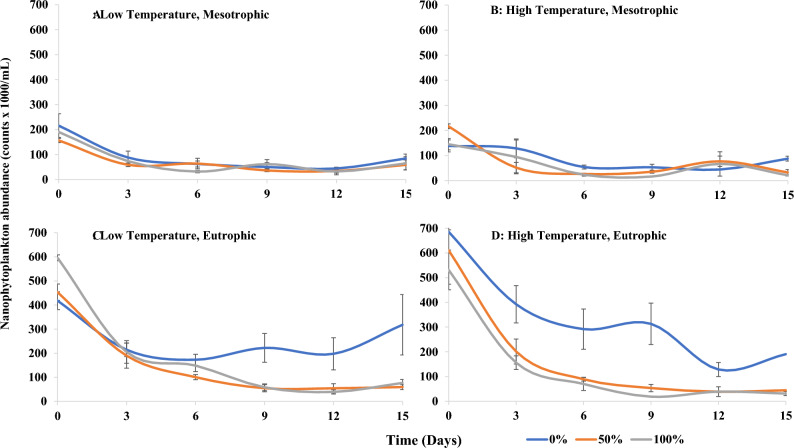
Spatio-temporal variability in nanophytoplankton abundance (mean ± SE) in (**A**) (low temperature, mesotrophic), (**B**) (high temperature, mesotrophic), (**C**) (low temperature, eutrophic) and (**D**) (high temperature, eutrophic) mesocosms at varying sandprawn densities (0%, 50% and 100%).

Variance in nanophytoplankton abundance was influenced by sandprawn density and eutrophication (main effects), as well as the interaction between (1) sandprawns and eutrophication and (2) sandprawns and temperature (Table 3). In contrast, variance in picophytoplankton abundance was not explained by any of the predictors tested (Table 3). Overall, trends in picophytoplankton abundance in response to predictor variables were unclear, in contrast to those for nanophytoplankton (Figs. 3 and 4). In mesotrophic conditions, variance in nanophytoplankton abundance among sandprawn densities was minor. Under eutrophic conditions, nanophytoplankton abundance was almost 3 times greater in control mesocosms by the end of the experiment relative to mesotrophic mesocosms. Under eutrophic conditions, nanophytoplankton abundance was reduced in the presence of sandprawns by approximately 3 times relative to controls (Fig. 4).

**Figure 5:**
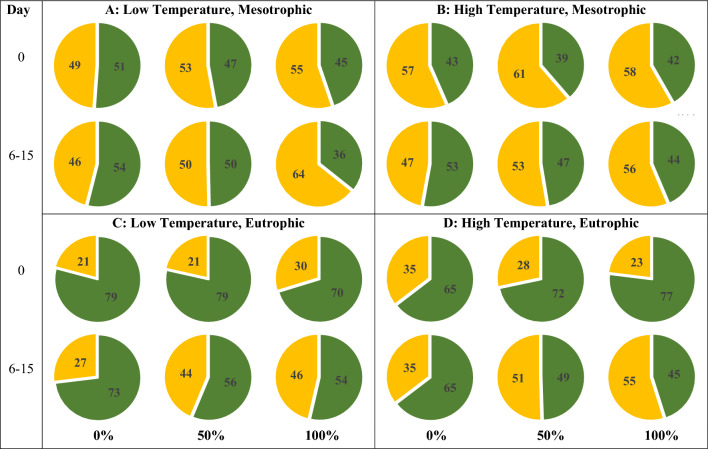
Changes in proportions of pico- (yellow) and nanophytoplankton (green) from the start of the experiment (Day 0) to Days 6–15 (averaged) in (**A**) (low temperature, mesotrophic), (**B**) (high temperature, mesotrophic), (**C**) (low temperature, eutrophic) and (**D**) (high temperature, eutrophic) mesocosms across varying sandprawn densities (0%, 50% and 100%).

Over the course of the experiment, eutrophication and sandprawn abundance elicited shifts in the composition of phytoplankton assemblages (Fig. 5). At the start of the experiment, contributions of picophytoplankton and nanophytoplankton to total phytoplankton abundance were similar, and negligibly changed in mesotrophic mesocosms over the course of the experiment, irrespective of temperature (Fig. 5). However, under eutrophic conditions, nanoplankton was more dominant at the start of the experiment (Day 0; 65–79%); this trend persisted to the end of the experiment in control mesocosm (Days 6–15; 65–73% nanophytoplankton). However, sandprawn presence reduced the dominance of nanophytoplankton under eutrophic conditions (54–65%) by the end of the experiment, resulting in more even contributions of pico- and nanophytoplankton (Fig. 5).

## Discussion

Interactions among top-down process, eutrophication/nutrient enrichment and warming have not received much attention in estuaries. However, in freshwater and open ocean ecosystems, research has emphasised the importance of understanding interactions among consumers, nutrients and warming for predicting ecological shifts via changes to phytoplankton assemblages^23–25,27–29,47,48^. Results from our mesocosm experiment highlight the potential for water-filtration by endobenthic sandprawns to prevent switches to eutrophic conditions by limiting phytoplankton blooms (including under high temperatures) and nanophytoplankton dominance. Therefore, appropriate management of sandprawn populations and habitats may assist in increasing resilience against algal blooms associated with coastal eutrophication in future.

By the end of our experiment, phytoplankton biomass declines in mesocosms containing sandprawns were consistent irrespective of temperature or eutrophication treatments. This suggests that levels of these global change predictors used were insufficient to impair sandprawn water filtration. Eutrophication has been suggested to increase the abundance of diseased organisms in marine ecosystems^22^. For example, an outbreak of a fecal-enteric microbe in the Florida Keys was linked to nutrient enrichment^49^, while aspergillosis (fungal infection) has been associated with declining water quality^50^. Aspergillotic lesions were shown to grow faster under higher nitrate levels^51^, which were also separately linked with the occurrence of black band disease in corals^52^. In our experiment, analysis of water column microbial assemblages using metagenomics indicated that relative to the mesotrophic treatments, eutrophic waters had twice the abundance of bacteria, with double the abundance of Enterobacterales, which includes taxa that are known to be pathogenic^53^ (Supplementary Table S3). Additionally, the near-doubling of phytoplankton biomass in eutrophic waters at the start of our experiment relative to mesotrophic mesocosms, and the subsequent shift towards very eutrophic states (approaching chl-*a* levels of 30ug/L) by the end of our experiment could have overwhelmed water filtration by sandprawns, given that these organisms are not obligate filter-feeders^25^. The resilience of sandprawn water filtration under eutrophic conditions therefore suggests minimal detrimental effects of potential toxins, pathogens, and high phytoplankton loads. Similarly, the high temperature treatment, which was roughly 4 degrees greater than maximum temperatures recorded near the sandprawn biotope in the Zandvlei Estuary, did not impair water filtration by sandprawns in our experiment. Visually, warming appeared to increase water filtration rate by sandprawns; under normal temperatures the rate of phytoplankton biomass decline in the mesocosms containing sandprawns saturated (point at which no further reductions were evident) by Day 6. In the warming treatment however, saturation was detected by Day 3. This may relate to the fact that sandprawns are ectothermic^54^, with warming increasing metabolic rate, thus causing an elevation in trophic resource (phytoplankton) demand. Similar effects have been reported for urchins, with top-down impact on macroalgae increasing at higher temperatures, in parallel with increases in urchin metabolic rate^17^. The robustness of sandprawn water filtration to high temperatures in our study is perhaps reflective of their wide geographical distribution along the South African coastline, wherein it occurs in both cool temperate and in sub-tropical regions^32^. In Durban Bay (sub-tropical east coast) mean summer water temperatures of 28.4 °C have been recorded in sandprawn habitats^55^, while in the Zandvlei Estuary, summer temperatures between 25.5 and 26 °C have been recorded near the sandprawn habitat in the lower reaches^35^ (Fig. 1). The robustness of sandprawn water filtration to temperatures as high as 29.5 °C in our experiment provides promising evidence of the resilience of sandprawns and filtration to future warming scenarios, at least in the context of populations within cool-temperate distribution ranges and temperature changes predicted up until 2100^31^.

Trait-shifts in phytoplankton have been associated with eutrophication, with larger size classes reported to dominate^56^ due to their competitive advantage in nutrient acquisition and conversion^57^. In our experiment, eutrophication did induce a phytoplankton shift to nanoplankton dominance, but this was opposed by sandprawn presence, resulting in even contributions of pico- and nanophytoplankton in mesocosms containing sandprawns under eutrophic conditions. Selective particle ingestion based on size and other traits has been reported for suspension feeders^58,59^. However, the effects we report regarding sandprawn-induced size-based shifts in phytoplankton under eutrophic conditions are not known for any deposit- or filter feeding endobenthic crustacean^25^, as far as we are aware. Importantly, shifts in phytoplankton size induced either through eutrophication or sandprawns may have important secondary implications for pelagic food web dynamics and trophic structure, given that phytoplankton size is an influential determinant of food web size and grazer traits^56^ ^and refs therein^.

The decline in phytoplankton biomass in mesocosms containing sandprawns in our experiment is likely a consequence of consumption. Research using stable isotope analysis concluded that particulate organic matter (POM) was the major dietary resource of sandprawns in the Gamtoos Estuary (Eastern Cape, South Africa^60^). However, given that sandprawns are not filter feeders^25^, phytoplankton consumption is likely achieved through cell adsorption on burrow walls, followed by sorting and ingestion. This idea is supported by Venter et al.^15^, who showed that sandprawn-induced phytoplankton declines occurred in parallel with enrichment of chl-*a* in burrow walls relative to sediment surfaces^15^. The estimate that a 1 m length of burrow of a filter-feeding endobenthic crustacean (*Upogebia pugettensis*) could indirectly filter 70% of total phytoplankton relative to direct filtration illustrates the phytoplankton filtration potential of endobenthic burrows^61^. This estimate takes on additional significance for deposit-feeding endobenthic crustaceans given the great depths to which they burrow (max 3.5–4 m^62,63^). While the major changes to phytoplankton biomass by sandprawns in our experiment can be ascribed to consumption, some effects may be due to abiotic changes induced by sandprawns, given that sandprawn density was linked statistically to changes in salinity, and pH (individually and/or interactively). However, we suggest that since abiotic change induced by sandprawns were small, they were unlikely to primarily have driven the phytoplankton responses recorded.

Phytoplankton biomass responses to eutrophication and warming in our experiment revealed interesting trends that may shed light on responses of coastal ecosystems to joint-warming and eutrophication in future. Eutrophic waters had double the chl-*a* biomass compared to non-eutrophic waters; this is expected given multiple studies linking high nutrient loading with phytoplankton proliferation^11,13,14^. However, over time, in the absence of sandprawns, eutrophic mesocosms at low temperature became more eutrophic (approaching chl-*a* levels of 30 ug/L), whereas warming suppressed phytoplankton biomass in the absence of sandprawns in non-eutrophic treatments, resulting in chl-*a* levels declining relative to start conditions. In the eutrophic treatments that lacked sandprawns, warming resulted in chl-*a* levels increasing, but not to levels recorded at low temperatures. These results suggest therefore that warming acted antagonistically to suppress eutrophication-induced phytoplankton proliferation. The suppressive role of warming that we detected corresponds to findings reported for open ocean ecosystems. While phytoplankton biomass has increased in some regions of the world’s oceans, at large scales, most observational and modelling evidence indicates that phytoplankton biomass and productivity have declined on average^64^. Ocean warming has been implicated as a driver, either through effects on stratification limiting nutrient supply and/or through modifying plankton metabolism^64^. It is important to note though that studies have suggested that harmful algal biomass may increase with warming in some marine and estuarine systems^65^.

Overall, our experiment has highlighted the resilience-conferring potential of sandprawns against eutrophication, including under warming conditions. However, care should be taken to avoid liberal extrapolation of our findings given that our approach was based on an ex situ experiment and that such experiments are typically conducted within a subset of natural conditions^66^. Further experimental, field and modelling studies are thus required to better understand water filtration by endobenthic crustaceans under an expanded set of environmental conditions. Our mesocosm approach allowed us to control confounding processes that are often prominent in heterogeneous and variable ecosystems such as estuaries^67,68^, enabling us to better understand cause-effect relationships among the complex set of predictor and response variables in our study. Given the (1) heterogeneous nature of estuaries and the potential for co-varying processes to mask cause-effect relationships, and (2) the impracticality in manipulating eutrophication and temperature levels in situ at meaningful scales to test pelagic responses, the use of ex situ experimentation in our study was appropriate in shedding light on a phenomenon (water filtration by sandprawns in a global change context) that we know little about. Broadly, small-scale ex situ experiments can be instrumental in improving understanding of larger-scale processes, including ecosystem responses to global change^69^. Mesocosm experimentation can also be useful in advancing understanding of ecological and global change processes occurring in microtidal, intermittently closed estuaries, given their shallow depths and limited flow, especially under closed conditions^27,28^.

At a global level, identifying processes that enhance ecosystem resilience against global change is a challenge for research, yet this is central to protecting ecosystem functions and services, including those on which humanity depends^47^. This idea underpins resilience-based ecosystem management, which entails understanding, managing, and conserving features of biological diversity that confer resilience to ecosystems^7,9,70^. In this context, our finding that sandprawns increase resilience against eutrophication, including under high temperatures, provide novel and promising evidence to support their inclusion in resilience-based ecosystem management. Our findings, along with the general robustness of endobenthic crustaceans to conditions such as hypoxia, hypercapnia and high sulphide levels^25^, suggests that their physiological and functional traits may be valuable in arresting future coastal degradation via eutrophication in future warming scenarios.

Central to the inclusion of sandprawns and similar organisms in resilience-based management practices is the need for identifying and managing processes that eradicate or weaken water-filtration functions, including coastal hardening (e.g. canalization), habitat destruction as part of coastal development, pollution and bait collecting^15^. Understanding how the multitude of coastal stressors impact water filtration by endobenthic crustaceans can provide critical support for ecosystem remediation, given the relevance of water quality to human health^71^. Integrating water filtration and endobenthic crustaceans can also raise public awareness of the functional importance of these organisms, so that their societal relevance can ascend beyond their current utility as bait organisms^25^. Even processes such as trampling of sandprawn habitats during recreational activity and/or bait collecting need to be understood insofar as impacts on water filtration are concerned, given the potential for these activities to impact burrow-builder populations, burrow superstructures and benthic environments^25,32^. The near 70% decline in sandprawn standing stocks in the Diep River Estuary (30 km north of the Zandvlei Estuary) between 1998 (40 million) and 2014 (12.3 million), as well as their range contraction that results in 93% of the population being exposed to bait collecting, raise concerns about the rapidity of sandprawn population declines in urban systems through multi-stressor interactions^72^. This research^72^ along with our experimental findings, flag a need to manage urban sandprawn populations to maximize their water filtration potential. Globally, managing the water-filtration capabilities of endobenthic crustacean populations may similarly assist in increasing resilience against coastal eutrophication in future. However, interventions aimed at managing nutrient inputs into estuarine systems should remain a priority.

### Supplementary Information

Supplementary Figure S1.

Supplementary Table S1.

Supplementary Table S2.

Supplementary Table S3.

## Data Availability

The datasets used and/or analyzed during the current study are available from the corresponding author on reasonable request.
