# Supplementary material for: Water filtration by endobenthic sandprawns enhances resilience against eutrophication under experimental global change conditions
Source: Sci Rep. 2023 Nov 4;13:19067. doi: 10.1038/s41598-023-46168-y (PMC10625564; doi:10.1038/s41598-023-46168-y)
Supplement: Supplementary file 1 — Supplementary Figure S1. [file 41598_2023_46168_MOESM1_ESM.docx]

Supplementary Figure S1: Irregular sediment topography created by dense *Kraussillichirus kraussi* (inset) populations in the sandprawn-dominated biotope (Site 1) in the Zandvlei Estuary. Holes on the sediment surface are burrow openings. The main image was taken 3 days after mouth opening, during which drainage into the Atlantic Ocean exposed parts of the benthic habitat. Image source Venter at al. 2020^15^.
