# Supplementary material for: Water filtration by endobenthic sandprawns enhances resilience against eutrophication under experimental global change conditions
Source: Sci Rep. 2023 Nov 4;13:19067. doi: 10.1038/s41598-023-46168-y (PMC10625564; doi:10.1038/s41598-023-46168-y)
Supplement: Supplementary file 2 — Supplementary Table S1. [file 41598_2023_46168_MOESM2_ESM.docx]

Supplementary Table 1: Spatio-temporal variability in water column temperature (Temp), salinity (Sal), pH, turbidity and dissolved oxygen (DO) levels across temperature, eutrophication and sandprawn density treatments over the 16-day mesocosm experiment. Means ± 1SE are shown.

| Treatment | Day | Sandprawn density | Temp (℃) | Sal | pH | Turbidity (NTU) | DO  (%) |
| --- | --- | --- | --- | --- | --- | --- | --- |
| **Low temp, mesotrophic** | 0 | 0 | 13.7±0.1 | 30.1±0.2 | 8±0.1 | 9±2.9 | 93.4±1.3 |
|  |  | 50 | 13.6±0 | 29.6±0.2 | 8±0 | 8.7±2.1 | 94.9±0.1 |
|  |  | 100 | 13.7±0.1 | 29.6±0.1 | 7.9±0 | 8.7±1.5 | 94.6±0 |
|  |  |  |  |  |  |  |  |
|  | 3 | 0 | 14.7±0.3 | 29.7±0.2 | 8.1±0.1 | 7.3±1.1 | 93±2 |
|  |  | 50 | 14.3±0.4 | 28.5±0.2 | 8±0.1 | 6.1±0.3 | 94.4±0.6 |
|  |  | 100 | 14.4±0.2 | 28.5±0 | 8.1±0.1 | 6.1±0.3 | 93.5±1.9 |
|  |  |  |  |  |  |  |  |
|  | 6 | 0 | 15.1±0.3 | 29.6±0.3 | 8.1±0 | 8.3±2.5 | 93.5±0.2 |
|  |  | 50 | 14.3±0.1 | 28.9±0.3 | 8.1±0 | 5.9±0.1 | 93.5±0.8 |
|  |  | 100 | 14.8±0.3 | 28.8±0 | 8.1±0.1 | 6.7±1 | 94.2±0.6 |
|  |  |  |  |  |  |  |  |
|  | 9 | 0 | 15.3±0.4 | 29.7±0.4 | 8.1±0 | 7.1±0.9 | 93.8±1.2 |
|  |  | 50 | 14.7±0.1 | 28.9±0 | 8.1±0 | 6.1±0.5 | 94.3±0.6 |
|  |  | 100 | 15.3±0.4 | 28.5±0.4 | 8.1±0 | 6.7±0.9 | 94±0.7 |
|  |  |  |  |  |  |  |  |
|  | 12 | 0 | 15.4±0.3 | 29.8±0.3 | 8.2±0.1 | 5.8±0 | 93.6±1.9 |
|  |  | 50 | 14.6±0.1 | 29.2±0 | 8.1±0.1 | 5.8±0.1 | 93.4±1.6 |
|  |  | 100 | 16.5±1.2 | 29.1±0.1 | 8.2±0.1 | 5.9±0.2 | 93.1±1.2 |
|  |  |  |  |  |  |  |  |
|  | 15 | 0 | 15.1±0.3 | 30.2±0.2 | 8.1±0 | 6±0.3 | 94.5±0.2 |
|  |  | 50 | 14.4±0.1 | 29.5±0.1 | 8.2±0.1 | 5.7±0 | 94.1±0.7 |
|  |  | 100 | 14.9±0.3 | 29.5±0 | 8.4±0.3 | 8.3±1.4 | 94.5±0.9 |
|  |  |  |  |  |  |  |  |
| **High temp, mesotrophic** | 0 | 0 | 13.9±0.3 | 30.3±0.1 | 8±0.1 | 8.1±1.3 | 95.1±0.5 |
|  |  | 50 | 13.8±0.2 | 30.3±0.2 | 8±0 | 10±1.6 | 94.4±0.3 |
|  |  | 100 | 13.8±0.2 | 30±0.4 | 8±0.1 | 8.8±2 | 95.3±0.7 |
|  |  |  |  |  |  |  |  |
|  | 3 | 0 | 30±1 | 32.1±0.2 | 8.3±0.1 | 7.8±1.5 | 97±0.6 |
|  |  | 50 | 28.2±0.8 | 30.8±0.5 | 8.3±0 | 6.1±0 | 95.4±0.3 |
|  |  | 100 | 27.8±0.4 | 29.8±0.6 | 8.3±0 | 6.7±0.3 | 96.8±0.5 |
|  |  |  |  |  |  |  |  |
|  | 6 | 0 | 29.7±0.5 | 33.8±0.6 | 8.3±0.1 | 6.7±0.3 | 96.9±0.6 |
|  |  | 50 | 28.7±0.4 | 32.3±0.7 | 8.3±0 | 8.7±1.9 | 96.6±0.3 |
|  |  | 100 | 28.6±0.2 | 30.8±1.2 | 8.2±0.1 | 6.5±0 | 96±0.8 |
|  |  |  |  |  |  |  |  |
|  | 9 | 0 | 29±0.5 | 34.9±0.9 | 8.4±0 | 6.6±0.4 | 96.9±0.3 |
|  |  | 50 | 29.1±0.8 | 33.6±0.8 | 8.4±0 | 6.5±0.4 | 96.7±0.9 |
|  |  | 100 | 28.7±0.3 | 31.6±1.5 | 8.3±0.1 | 6.6±0.1 | 98.5±1 |
|  |  |  |  |  |  |  |  |
|  | 12 | 0 | 29±0.3 | 35.8±1.2 | 8.4±0 | 6.2±0.1 | 101.5±1.3 |
|  |  | 50 | 29.5±0.7 | 34.2±0.8 | 8.3±0 | 6.4±0.2 | 98.9±0.5 |
|  |  | 100 | 28.6±0.3 | 32.5±1.4 | 8.3±0 | 6.2±0.1 | 97.5±0.5 |
|  |  |  |  |  |  |  |  |
|  | 15 | 0 | 29.4±0.3 | 36.6±1.1 | 8.4±0 | 6.9±0.7 | 97.3±0.5 |
|  |  | 50 | 29.4±0.7 | 35.4±0.9 | 8.3±0 | 6.4±0.2 | 96.9±0.1 |
|  |  | 100 | 28.8±0.1 | 33.6±1.6 | 8.3±0 | 6.7±0.6 | 97.2±0.4 |
| **Low temp,**  **eutrophic** | 0 | 0 | 13.7±0.2 | 29.5±0.1 | 8±0 | 6.2±0.1 | 95.3±0.1 |
|  |  | 50 | 13.6±0.1 | 29.3±0.2 | 8±0 | 8.7±1.2 | 94.9±0.2 |
|  |  | 100 | 13.6±0.1 | 29.5±0.2 | 8±0.1 | 8.9±2.1 | 93.9±1 |
|  |  |  |  |  |  |  |  |
|  | 3 | 0 | 14±0.1 | 28.8±0.2 | 8±0 | 6±0.2 | 95.8±0.5 |
|  |  | 50 | 14±0.3 | 27.9±0.2 | 8.1±0.1 | 5.9±0.1 | 93.6±1.1 |
|  |  | 100 | 14.9±0.3 | 28.3±0.4 | 8.2±0.1 | 5.9±0.1 | 92.8±0.7 |
|  |  |  |  |  |  |  |  |
|  | 6 | 0 | 14.5±0.1 | 28.7±0.3 | 8.1±0.1 | 6.9±0.4 | 93.8±1.2 |
|  |  | 50 | 14.3±0.4 | 28.2±0.2 | 8.1±0.1 | 5.9±0.2 | 93.4±0.8 |
|  |  | 100 | 15.1±0.4 | 28.3±0.1 | 8.1±0 | 6.5±0.5 | 94.4±0.1 |
|  |  |  |  |  |  |  |  |
|  | 9 | 0 | 14.9±0.1 | 28.6±0.1 | 8.1±0 | 6±0.1 | 94.3±1 |
|  |  | 50 | 14.5±0.3 | 28.3±0.3 | 8.1±0 | 5.8±0 | 94.5±0.1 |
|  |  | 100 | 15.5±0.4 | 28.6±0.2 | 8.1±0 | 5.8±0.1 | 94.6±0 |
|  |  |  |  |  |  |  |  |
|  | 12 | 0 | 14.8±0.1 | 28.8±0.1 | 8.1±0 | 5.8±0 | 95.2±0.1 |
|  |  | 50 | 14.5±0.3 | 28.7±0.2 | 8.1±0.1 | 5.7±0 | 92.2±2 |
|  |  | 100 | 15.5±0.6 | 28.7±0.2 | 8.2±0.1 | 5.8±0.1 | 94.1±0.8 |
|  |  |  |  |  |  |  |  |
|  |  |  |  |  |  |  |  |
|  | 15 | 0 | 14.8±0.2 | 29.1±0.2 | 8.1±0 | 6.9±1 | 94.3±0.6 |
|  |  | 50 | 14.3±0.3 | 29±0.3 | 8.1±0 | 5.8±0.1 | 94.5±0.4 |
|  |  | 100 | 15.1±0.4 | 29.1±0.2 | 8.1±0 | 6.8±1 | 94.4±0.8 |
|  |  |  |  |  |  |  |  |
| **High temp,**  **eutrophic** | 0 | 0 | 13.6±0 | 29.1±0.1 | 8±0 | 10.7±0 | 95±0.3 |
|  |  | 50 | 13.5±0 | 29.4±0 | 8±0 | 8.7±1.6 | 94.9±0.3 |
|  |  | 100 | 13.5±0.1 | 29.5±0.2 | 8±0 | 9.4±1.8 | 94.9±0.4 |
|  |  |  |  |  |  |  |  |
|  | 3 | 0 | 28.8±0.5 | 30.3±0.7 | 8.3±0 | 6.6±0.3 | 95.7±0.6 |
|  |  | 50 | 27.8±0.6 | 29.5±0.2 | 8.3±0.1 | 6.5±0.1 | 97.8±0.9 |
|  |  | 100 | 26.8±0.4 | 29±0.1 | 8.3±0.1 | 6.2±0 | 96.1±0.4 |
|  |  |  |  |  |  |  |  |
|  | 6 | 0 | 29.6±0.7 | 31.1±0.4 | 8.4±0.1 | 7.6±0.7 | 95.9±0.6 |
|  |  | 50 | 29±0.5 | 36.8±5.5 | 8.4±0.1 | 6.6±0.1 | 96.2±0.5 |
|  |  | 100 | 27.4±0.2 | 30.4±0.1 | 8.3±0.1 | 6.3±0 | 96.1±0.8 |
|  |  |  |  |  |  |  |  |
|  | 9 | 0 | 29.3±0.8 | 31.6±0.5 | 8.4±0.1 | 6.3±0.1 | 96.6±0.4 |
|  |  | 50 | 29±0.2 | 32.4±0.1 | 8.4±0 | 6.3±0.1 | 96.5±0.2 |
|  |  | 100 | 28±0.2 | 31.6±0.2 | 8.3±0 | 6.2±0.1 | 97.8±0.4 |
|  |  |  |  |  |  |  |  |
|  | 12 | 0 | 28.9±0.4 | 32.7±0.7 | 8.4±0 | 6.5±0.1 | 99±1.4 |
|  |  | 50 | 28.8±0.4 | 33.9±0.3 | 8.4±0 | 6.5±0.3 | 97.1±0.5 |
|  |  | 100 | 27.8±0.2 | 32.9±0.4 | 8.3±0 | 6.4±0.2 | 100.2±2.2 |
|  |  |  |  |  |  |  |  |
|  | 15 | 0 | 28.8±0.1 | 33.8±0.8 | 8.4±0 | 7±0.7 | 97.7±0 |
|  |  | 50 | 29.4±0.3 | 34.8±0.4 | 8.3±0 | 6.2±0.1 | 96.1±0.3 |
|  |  | 100 | 28.1±0.2 | 34.1±0.4 | 8.2±0 | 6.2±0.1 | 97.8±0.9 |
