# Supplementary material for: Water filtration by endobenthic sandprawns enhances resilience against eutrophication under experimental global change conditions
Source: Sci Rep. 2023 Nov 4;13:19067. doi: 10.1038/s41598-023-46168-y (PMC10625564; doi:10.1038/s41598-023-46168-y)
Supplement: Supplementary file 3 — Supplementary Table S2. [file 41598_2023_46168_MOESM3_ESM.docx]

Supplementary Table S2: Spatio-temporal variability in pelagic nutrient (phosphate, ammonium, nitrate, nitrite) concentrations across temperature, eutrophication and sandprawn density treatments over the 16-day mesocosm experiment. Means ± 1SE are shown.

|  |  |  |  |  |  |  |
| --- | --- | --- | --- | --- | --- | --- |
| **Treatment** | **Day** | **Sandprawn density** | **Phosphate (PO_4_^3-^) (mg/L)** | **Ammonium (NH_4_^±^)**  **(mg/L)** | **Nitrate**  **(NO_3_^-^)**  **(mg/L)** | **Nitrite (NO_2_^-^)**  **(mg/L)** |
| **Low temp, mesotrophic** | 0 | 0 | 3.1±1.0 | 5.7±3.4 | 4.0±4.0 | 0.1±0.0 |
|  |  | 50 | 4.4±0.3 | 7.6±1.3 | 0.0±0.0 | 0.1±0.0 |
|  |  | 100 | 2.9±0.9 | 2.7±0.8 | 0.7±0.7 | 0.1±0.1 |
|  |  |  |  |  |  |  |
|  | 7 | 0 | 3.1±0.6 | 4.5±3.1 | 0.7±0.7 | 0.1±0.0 |
|  |  | 50 | 2.5±0.3 | 9.2±1.8 | 0.6±0.6 | 0.1±0.0 |
|  |  | 100 | 2.5±1.1 | 10.1±2.2 | 0.3±0.3 | 0.1±0.0 |
|  |  |  |  |  |  |  |
|  | 13 | 0 | 1.6±0.2 | 8.4±3.3 | 0.0±0.0 | 0.1±0.0 |
|  |  | 50 | 2.7±0.4 | 2.9±0.4 | 0.5±0.5 | 0.1±0.0 |
|  |  | 100 | 3.0±0.2 | 6.4±3.2 | 0.0±0.0 | 0.1±0.1 |
|  |  |  |  |  |  |  |
| **High temp, mesotrophic** | 0 | 0 | 3.3±1.4 | 8.3±1.0 | 1.0±1.0 | 0.1±0.0 |
|  |  | 50 | 1.8±0.5 | 6.6±3.3 | 0.0±0.0 | 0.1±0.0 |
|  |  | 100 | 2.7±0.8 | 7.2±3.4 | 1.1±0.5 | 0.1±0.0 |
|  |  |  |  |  |  |  |
|  | 7 | 0 | 2.6±1.0 | 10.2±2.0 | 0.8±0.5 | 0.5±0.2 |
|  |  | 50 | 3.2±0.1 | 10.1±1.4 | 1.9±1.9 | 0.4±0.1 |
|  |  | 100 | 2.8±0.6 | 11.3±0.7 | 0.1±0.1 | 0.1±0.0 |
|  |  |  |  |  |  |  |
|  | 13 | 0 | 2.0±0.2 | 11.5±1.1 | 11±5.4 | 2.7±0.6 |
|  |  | 50 | 2.3±0.4 | 6.2±3.0 | 10.1±3.6 | 2.0±0.6 |
|  |  | 100 | 1.6±0.6 | 8.7±3.5 | 7.0±1.8 | 1.7±0.5 |
|  |  |  |  |  |  |  |
| **Low temp, eutrophic** | 0 | 0 | 3.6±0.5 | 8.0±3.1 | 0.0±0.0 | 0.1±0.0 |
|  |  | 50 | 3.8±0.5 | 6.8±3.2 | 1.3±1.3 | 0.0±0.0 |
|  |  | 100 | 3.7±0.4 | 4.0±3.3 | 1.8±1.8 | 0.1±0.0 |
|  |  |  |  |  |  |  |
|  | 7 | 0 | 3.2±0.2 | 2.7±2.2 | 1.2±0.8 | 0.1±0.0 |
|  |  | 50 | 3.2±0.7 | 6.1±2.1 | 0.0±0.0 | 0.1±0.0 |
|  |  | 100 | 3.2±0.7 | 6.2±2.6 | 0.0±0.0 | 0.1±0.0 |
|  |  |  |  |  |  |  |
|  | 13 | 0 | 2.7±0.5 | 10±1.1 | 2.3±1.4 | 0.1±0.0 |
|  |  | 50 | 2.3±0.6 | 6.3±3.2 | 0.0±0.0 | 0.2±0.0 |
|  |  | 100 | 2.5±0.5 | 5.4±2.7 | 0.0±0.0 | 0.1±0.0 |
|  |  |  |  |  |  |  |
| **High temp, eutrophic** | 0 | 0 | 2.5±1.1 | 10.3±0.6 | 0.0±0.0 | 0.0±0.0 |
|  |  | 50 | 1.8±0.5 | 5.5±3.6 | 1.6±1.6 | 0.0±0.0 |
|  |  | 100 | 0.5±0.3 | 6.8±3.5 | 0.4±0.4 | 0.0±0.0 |
|  |  |  |  |  |  |  |
|  | 7 | 0 | 1.9±0.3 | 10.5±1.0 | 2.4±1.2 | 0.3±0.0 |
|  |  | 50 | 2.3±1.0 | 10.4±0.6 | 3.5±0.8 | 0.4±0.1 |
|  |  | 100 | 3.4±0.3 | 5.2±2.1 | 1.8±1.0 | 0.1±0.0 |
|  |  |  |  |  |  |  |
|  | 13 | 0 | 1.8±0.4 | 6.8±3.2 | 11.8±7.2 | 3.2±2.0 |
|  |  | 50 | 1.8±0.4 | 12±0.4 | 11.1±5.2 | 3.8±1.1 |
|  |  | 100 | 3.3±0.3 | 11.8±0.6 | 1.2±0.7 | 0.3±0.1 |
