# Supplementary material for: Water filtration by endobenthic sandprawns enhances resilience against eutrophication under experimental global change conditions
Source: Sci Rep. 2023 Nov 4;13:19067. doi: 10.1038/s41598-023-46168-y (PMC10625564; doi:10.1038/s41598-023-46168-y)
Supplement: Supplementary file 4 — Supplementary Table S3. [file 41598_2023_46168_MOESM4_ESM.docx]

Supplementary Table S3: Results of SIMPER analysis identifying taxa that contributed most (70% cumulatively) to differentiating bacterial assemblages between mesotrophic and eutrophic waters at the end of the 16-day mesocosm experiment. Bacterial taxa and 16S gene abundances were quantified from single 1L water samples from control mesocosms (low temperature, no sandprawns). Samples were sequenced using a Minion Mk1C (Oxford Nanopore Technologies) and analysed using EMU v3^1^ using the SILVA v1.38.1 database. SIMPER was carried out in PRIMER v7 (Plymouth Routines in Multivariate Ecological Research). Bacterial orders are presented in parentheses next to genus names.

|  | **Mesotrophic** | **Eutrophic** |  |  |  |  |
| --- | --- | --- | --- | --- | --- | --- |
| **Taxon** | **Ave**  **Abundance** | **Ave**  **Abundance** | **Ave**  **Dissimilarity** | **Diss/**  **SD** | **Contribution (%)** | **Cumulative (%)** |
| *Citrobacter braakii* (Enterobacterales) | 454.67 | 24179.67 | 13.19 | 0.70 | 16.04 | 16.04 |
| *Enterobacter cloacae* (Enterobacterales) | 10120.67 | 17012 | 10.21 | 0.95 | 12.41 | 28.44 |
| *Raoultella planticola* (Enterobacterales) | 10318 | 1917.67 | 4.83 | 0.77 | 5.87 | 34.31 |
| *Citrobacter werkmanii* (Enterobacterales) | 86 | 6475 | 2.42 | 0.73 | 2.94 | 37.25 |
| *Pseudomonas* x. (Pseudomonadales) | 4982.33 | 132.33 | 2.36 | 0.66 | 2.87 | 40.12 |
| *Delftia*_x. (Burkholderiales) | 45.67 | 5525.67 | 2.06 | 0.67 | 2.50 | 42.62 |
| *Gracilibacteria*_x. (Candidatus) | 736.67 | 5118 | 1.96 | 0.84 | 2.39 | 45.01 |
| *Klebsiella pneumoniae* (Enterobacterales) | 790 | 3172.33 | 1.41 | 1.31 | 1.71 | 46.72 |
| *Klebsiella*_x (Enterobacterales) | 2595.67 | 128.67 | 1.34 | 0.67 | 1.62 | 48.34 |
| *Stenotrophomonas*_x (Xanthomonadales) | 99.33 | 3050.67 | 1.24 | 1.23 | 1.51 | 49.85 |
| *Aestuariicoccus*_x (Rhodobacterales) | 1700.33 | 2480.67 | 1.22 | 1.09 | 1.48 | 51.34 |
| *Kluyvera georgiana* (Enterobacterales) | 2610 | 235.33 | 1.19 | 0.84 | 1.45 | 52.79 |
| *Escherichia-Shigella coli* (Enterobacterales) | 833.33 | 2465.33 | 1.17 | 0.90 | 1.42 | 54.21 |
| *Holosporaceae*_x (Holosporales) | 19.33 | 3105 | 1.16 | 0.68 | 1.40 | 55.61 |
| *Gammaproteobacteria* x (Enterobacterales) | 192.67 | 3066 | 1.13 | 0.72 | 1.38 | 56.99 |
| *Serratia marcescens* (Enterobacterales) | 615.67 | 2803 | 1.13 | 1.48 | 1.37 | 58.36 |
| *Citrobacter pasteurii* (Enterobacterales) | 2176.67 | 397.33 | 1.06 | 0.84 | 1.28 | 59.65 |
| *Edwardsiella ictaluri* (Enterobacterales) | 1330.33 | 1745 | 1.05 | 0.95 | 1.28 | 60.93 |
| *Stenotrophomonas maltophilia* (Xanthomonadales) | 286.33 | 2096.33 | 1.05 | 0.86 | 1.28 | 62.20 |
| *Serratia*_x (Enterobacterales) | 2297.33 | 36.33 | 1.03 | 0.67 | 1.25 | 63.45 |
| *Enterobacter*_x (Enterobacterales) | 1931.67 | 133.67 | 0.90 | 0.68 | 1.09 | 64.55 |
| *Enterobacter kobei* (Enterobacterales) | 107.67 | 1703.67 | 0.86 | 0.69 | 1.05 | 65.59 |
| *Mitochondria*_x (Rickettsiales) | 1788 | 118.33 | 0.80 | 0.70 | 0.97 | 66.56 |
| NS3aNS3a x (Flavobacteriales) | 66.33 | 2055 | 0.79 | 0.94 | 0.96 | 67.52 |
| *Marivita*_x (Rhodobacterales) | 224 | 1386.33 | 0.71 | 0.76 | 0.87 | 68.38 |
| *Marivita lacus* (Rhodobacterales) | 352.67 | 1463.33 | 0.71 | 0.85 | 0.86 | 69.24 |
| *Citrobacter*_x (Enterobacterales) | 1385 | 1516.67 | 0.66 | 1.12 | 0.81 | 70.05 |
|  |  |  |  |  |  |  |
| Mean bacterial abundance | 48146.34 | 93519.33 |  |  |  |  |
| Mean Enterobacterales abundance | 37845.35 | 66987.67 |  |  |  |  |

**Reference**

1. Curry, K.D., Wang, Q., Nute, M.G. et al. Emu: species-level microbial community profiling of full-length 16S rRNA Oxford Nanopore sequencing data. Nat Methods 19, 845–853 (2022).
